# Danggui Buxue Tang Attenuates Tubulointerstitial Fibrosis via Suppressing NLRP3 Inflammasome in a Rat Model of Unilateral Ureteral Obstruction

**DOI:** 10.1155/2016/9368483

**Published:** 2016-10-31

**Authors:** Linna Wang, Jiwei Ma, Cunxia Guo, Changyong Chen, Zhong Yin, Xueguang Zhang, Xiangmei Chen

**Affiliations:** ^1^Department of Nephrology, Chinese PLA General Hospital, Chinese PLA Institute of Nephrology, State Key Laboratory of Kidney Diseases, National Clinical Research Center for Kidney Diseases, Beijing 100853, China; ^2^Department of Nephrology, Henan Province Hospital of Chinese Medicine, Zhengzhou, Henan 450002, China; ^3^The First Affiliated Hospital of Henan Traditional Chinese Medicine University, Zhengzhou, Henan 450008, China; ^4^Department of Radiology, Xiangya Hospital, Central South University, Changsha, Hunan 410008, China

## Abstract

Inflammation significantly contributes to the progression of chronic kidney disease (CKD). This study aimed to characterize Danggui Buxue Tang (DBT) renoprotection and relationship with NOD-like receptors family pyrin domain-containing 3 (NLRP3) inflammasome expression in rats with unilateral ureteral obstruction (UUO). Sprague-Dawley rats were subjected to UUO and randomly assigned to untreated UUO, enalapril-treated (10 mg/kg/day), and DBT-treated (9 g/kg/day) groups. Sham-operated rats served as controls, with 8 rats in each group. All rats were sacrificed for blood and renal specimen collection at 14 days after UUO. Untreated UUO rats exhibited azotemia, intense tubulointerstitial collagen deposition, upregulations of tubulointerstitial injury index, augmentation levels of collagen I (Col I), *α*-smooth muscle actin (*α*-SMA), NLRP3, apoptosis-associated speck-like protein containing a caspase recruitment domain (ASC), pro-caspase-1, caspase-1, IL-1*β*, and pro-IL-1*β*. DBT treatment significantly attenuated interstitial collagen deposition and tubulointerstitial injury, lowering Col I and *α*-SMA levels. Synchronous expressions of NLRP3, ASC, pro-caspase-1, caspase-1, pro-IL-1*β*, and IL-1*β* decreased in renal tissue. In comparison to enalapril, DBT significantly reduced tubulointerstitial injury, interstitial collagen deposition, and expressions of Col I and IL-1*β*. Thus, DBT offers renoprotection in UUO rats, which was associated with suppressing NLRP3 inflammasome expression and following reduction of the secretion of cytokine IL-1*β*. The mechanisms of multitargets of traditional Chinese medicine can be better used for antifibrotic treatment.

## 1. Introduction

Fibrosis is the common final manifestation of progressive diseases in kidney, lung, heart, and liver that leads to end-stage organ diseases. With the increasing patients and limited therapeutic options, chronic renal disease (CKD) and tubulonterstitial fibrosis (TIF) are becoming an important cause of morbidity and mortality worldwide. TIF involves an excess accumulation of interstitial extracellular matrix and myofibroblasts accompanied by tubule atrophy.

Inflammation significantly contributes to the progression of chronic kidney disease (CKD) and TIF [1–4]. Inflammasomes are multiprotein cytoplasmic complexes that serve as pattern recognition receptors and regulate proinflammatory cytokine Interleukin-1*β* (IL-1*β*) production [5]. NOD-like receptors family pyrin domain-containing 3 (NLRP3), the most characterized inflammasome, forms complexes comprised of adaptor proteins such as the apoptosis-associated speck-like protein containing a caspase recruitment domain (ASC) and the serine protease caspase-1 (Casp1). NLRP3 inflammasome triggers caspase-1 activation and IL-1*β* maturation in response to diverse stimuli in a murine model of unilateral ureteral obstruction (UUO) [6–8]. IL-1*β* (p17) is synthesized as an inactive precursor molecule (pro-IL-1*β*, p35) by cells of the innate immune system and is a proinflammatory cytokine produced by activated macrophages and monocytes. Infiltrating macrophages are the major source of IL-1*β* in renal disease.

Other proteins forming inflammasomes include the NLRs, NLRP1, NLRC4 (Ipaf-1), and the human interferon-inducible 200 protein AIM2 (absent in melanoma-2) [9]. NLRC4 can activate caspase-1 in the absence of the adaptor ASC [10, 11]. NLRP1 interacts directly with caspase-1 and caspase-5. AIM2 acts as a receptor that is activated by DNA binding. Komada et al. reported that the NLRC4 and AIM2 inflammasomes were not involved in the pathophysiological characteristics of UUO [12]. Other NLRs such as NLRP2 and NLRP12 have also been shown to interact with ASC and activate caspase-1 only* in vitro*; however, the biologic significance of these observations and whether these NLRs form true inflammasomes are unclear [13, 14].

Inflammatory process in CKD is closely related to the production of proinflammatory cytokines, such as tumor necrosis factor-*α* (TNF-*α*), IL-1*β*, and IL-6. IL-1*β* is a potent proinflammatory and profibrogenic cytokine, and its overproduction is associated with chronic inflammation and the development of pathologies such as gout, diabetes, or cancer [15, 16]. IL-1*β* induces human proximal tubule cells injury, tubular epithelial-myofibroblast transdifferentiation (TEMT), *α*-smooth muscle actin (*α*-SMA) expression, fibronectin production, and renal fibrosis through a TGF-*β*1-dependent mechanism [17, 18]. Intervention therapies assigned to the blockade of proinflammatory cytokines are considered potential treatment agents for CKD patients. Since many plants contain some compounds with anti-inflammatory activity, their consumption may be able to prevent the development of inflammatory-based diseases.

Danggui Buxue Tang (DBT) was formulated originally during the Jin dynasty (1247 AD) of China, with two main ingredients, Danggui (*Radix Angelicae Sinensis, RAS*) and Huangqi (*Radix Astragali, RA*), with a weight ratio of 1 : 5 [19]. The combination of Radix Angelicae Sinensis and Radix Astragali was demonstrated to possess a renoprotective effect on animal models of CKD [20–24]. DBT* per se* alleviated the progression of diabetic nephropathy induced with streptozotocin (STZ) [21, 22] and liver fibrosis induced with CCl(4) and high-fat food [25]. Furthermore, total glucosides of DBT (DBTG), extract from DBT, had been confirmed to have the ability of attenuating bleomycin-induced pulmonary fibrosis [26, 27].

In the present study, we hypothesize that DBT attenuates renal TIF through regulating the level of IL-1*β* by inhibiting NLRP3 inflammasome expression and activation. The results of the present study may explore other potential mechanisms of DBT and provide the basis for the clinical treatment of TIF.

## 2. Materials and Methods

### 2.1. Animals and Experimental Protocol

A total of 32 male Sprague-Dawley rats (8 weeks old, 180–200 g) were purchased from experimental animal center of Henan province (2005-0012 SYXK, Zhengzhou, China). All animals were housed in plastic cages with a room temperature of 22 ± 1°C and relative humidity of 50 ± 20% and under a 12-h light/dark cycle. The studies were performed in accordance with the guidelines for the humane treatment of animals as set forth by the Association of Laboratory Animal Sciences and the Center for Laboratory Animal Sciences at Medical School of Zhengzhou University. Unilateral ureter obstruction was induced as described previously [28]. This study was approved by the ethics committee of Medical School of Zhengzhou University.

All rats received preoperative analgesia (intraperitoneal injection of 50 mg/kg pentobarbital) and the right ureters were subsequently ligated with 6.0 silk through a small abdominal incision under 10% chloral hydrate-induced anesthesia. The abdomen was closed in two layers and rats were allowed to recover from surgery for 12 hours at 28°C in a ventilated stove. Immediately after the surgery, rats were randomly assigned into four groups; sham-operated group, serving as control, underwent a sham operation consisting of laparotomy and manipulation of the renal pedicles but without damage to the kidney for normal controls. Three groups of UUO rats were subjected to vehicle, enalapril (10 mg/kg/day, Yangtze River Pharmaceutical Co., Ltd.), or DBT (9 g/kg/day) administration, named as UUO group, enalapril group, or DBT group, respectively, with 8 rats in each group. Rats were sacrificed 14 days after surgery. Rat serum was collected for urea nitrogen and creatinine determination. The obstructed kidney underwent coronal incision, half of the kidney were fixed in 10% neutral buffered formalin for 12 hours and then paraffin embedding. The remaining renal cortex were frozen in liquid nitrogen for further use.

### 2.2. Plant Materials and DBT Preparation

DBT consists of RAS and RA at a 1 : 5 ratio. The herbs originated from Neimeng and Gansu province, China. Slices of the herbs were purchased from the Herbal Pharmacies of Henan Province Hospital of Chinese Medicine and were extracted twice. The extraction process of the crude drugs was performed under strict quality control. RAS (200 g) and RA (1000 g) were first boiled together in 6x volume of water for 1 h, and the residue from first extraction was boiled in 8x volume of water for 1.5 h. Finally, the aqueous extracts were combined, filtered to remove insoluble debris, and stored at −20°C. According to the dose conversion coefficient tables of animals and human and the data of preliminary experimental study, the rat daily dose is equal to the content of RAS 1.5 g/kg/d and RA 7.5 g/kg/d.

### 2.3. Renal Pathology and Immunohistochemistry Staining

For histopathological analysis, 4 *μ*m thick renal sections were cut from the paraffin blocks. The hematoxylin-eosin- (HE-) stained sections were evaluated semiquantitatively, as described previously, to assess renal tubulointerstitial injury [29]. To further assess the degree of tubulointerstitial collagen deposition, Masson's trichrome staining sections were graded as mentioned before [30]. Immunohistochemistry staining was processed using DAKO EnVision System (Dako Diagnostics, Zug, Switzerland) for renal immunohistochemical assay. The slides were incubated with a primary antibody against collagen I (1 : 500, Abcam, Cambridge, UK). Given the homogeneity of the target proteins' staining, the interstitial staining of type I collagen was measured as described by a blinded observer using computerized morphometry (Image-Pro Plus 6.0 software, Media Cybernetics, Bethesda, MD) [31].

### 2.4. Western Blots Analysis

Kidney cortex tubular tissues were washed with phosphate buffer solution (PBS) and lysed in a buffer containing 20 mmol/L Tris-HCl (pH 7.4), 4% sodium dodecyl sulfate (SDS), 10% glycerol, and a cocktail of protease inhibitors. Twenty micrograms of lysate proteins was separated by sodium dodecyl sulfate polyacrylamide gel electrophoresis (SDS-PAGE) under reducing conditions and electroblotted onto polyvinylidene difluoride (PVDF) membranes (Millipore, USA). The membranes were incubated immersed in a blocking solution composed of 5% nonfat dry milk and TBS-T (0.05% Tween 20, 20 mmol/L Tris-HCl, and 150 mmol/L NaCl, pH 7.6) for 1 h at room temperature, then incubated with individual primary antibodies at 4°C overnight. Horseradish peroxidase-labeled secondary antibodies were added for 1 hour at room temperature after membranes were washed with TBS-T for three times. Signals were developed using the enhanced chemiluminescence reagents (ECL, Amersham Biosciences) plus Western blotting detection reagents (GE Healthcare, UK) and X-ray film (Kodak, USA). Bands were visualized with enhanced chemiluminescence and quantified using ImageJ. Primary antibodies used were NLRP3 (1 : 1000, Cell Signaling Technology, Boston, MA), ASC (1 : 1000, Enzo Life Sciences, Inc., Farmingdale, NY), anti-pro-caspase-1 (1 : 1000, Cell Signaling Technology, Boston, MA), anti-cleaved-caspase-1 (1 : 1000, Cell Signaling Technology, Boston, MA), anti-pro-IL-1*β* (1 : 1000, Santa Cruz Biotechnology (Shanghai) Co., Ltd.), anti-cleaved-IL-1*β* (1 : 1000, Cell Signaling Technology, Boston, MA), and *β*-tubulin (1 : 1000, Cell Signaling Technology, Boston, MA).

### 2.5. RNA Extraction and Real-Time PCR Quantitation

Total RNA was isolated from kidney tissue using TRIzol Reagent according to the manufacturer's instructions (Invitrogen Corporation, Carlsbad, CA). For quantitative real-time PCR analysis, complimentary DNA (cDNA) was synthesized from 1 *μ*g of total RNA using a RevertAid First Strand cDNA Synthesis Kit (Thermo Scientific Fermentas, Thermo Fisher Scientific Inc., Waltham, MA) and analyzed using SYBR Green PCR reagent kit (SYBR PremixEx Taq II, Takara, Japan) in CFX96 Real-Time System (BIO-RAD Laboratories, Hercules, CA). The specific primers (collagen I: upstream 5′-*TCAGGGGCGAAGGCAACAGT*-3′ and downstream 5′-*TTGGGATGGAGGGAGTTTACACGA*-3′; NLRP3: upstream 5′-*CCCCGTGAGTCCCATTA*-3′ and downstream 5′-*GACGCCCAGTCCAACAT*-3′; ASC: upstream 5′-*GAAGCTGCTGACAGTGCAAC*-3′ and downstream 5′-*GCCACAGCTCCAGACTCTTC*-3′; caspase-1: upstream 5′-*TGCCTGGTCTTGTGACTTGGAG*-3′ and downstream 5′-*ATGTCCTGGGAAGAGGTAGAAACG*-3′; IL-1*β*: upstream 5′-*GCACTACAGGCTCCGAGATGAA*-3′ and downstream 5′-*GTCGTTGCTTGGTTCTCCTTGT*-3′; and *β*-actin: upstream 5′-*GGCCAACCGTGAAAAGATGA*-3′ and downstream 5′-*GACCAGAGGCATACAGGGACAA*-3′) were designed from the GenBank sequences and synthesized by Bio Basic (Generay Biotechnology, Shanghai, China). All primer sequences were checked in GenBank to avoid inadvertent sequence homologies. The quantity of mRNA was calculated based on the cycle threshold (CT) values which were standardized with the amount of the housekeeping gene *β*-actin. ΔCT was the value from the corresponding target gene's CT value subtracting the CT value of the *β*-actin. Further calculation was performed using 2^−ΔΔCT^ method, and the results were expressed as the *n*-fold difference relative to normal controls.

### 2.6. Enzyme-Linked Immunosorbent Assay (ELISA) for Cytokines IL-1*β*

For further confirmation of the production of IL-1*β* in obstructive renal tissue, IL-1*β* cytokines were measured in kidney homogenates using commercial ELISA kits (Invitrogen, Grand Island, NY), following the protocol provided by the manufacturer.

### 2.7. Statistical Analysis

All data were expressed as means ± standard deviation, and were analyzed with SPSS 18.0 software (SPSS Inc., Ill., USA). One-way analysis of variance (ANOVA) was performed to estimate the statistical significance of difference among the groups. when there was a significant difference determined by ANOVA, multiple-comparison tests were applied. Significance was accepted when *p* < 0.05 and *p* < 0.01.

## 3. Results

### 3.1. DBT Offers Improved Renal Protection in Rats with UUO at 14 Days

**Figure 1:**
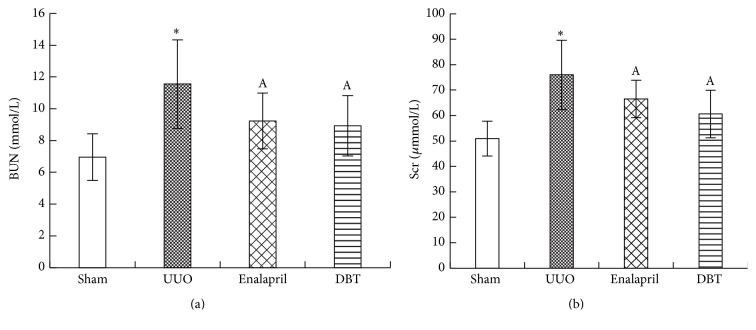
Levels of serum creatinine (Scr) and blood urea nitrogen (BUN) in each group. BUN was significantly higher in UUO rats compared with sham-operated rats and was lower in enalapril group and DBT group, compared with UUO group. There was no difference between enalapril-treated rats and DBT-treated rats (a). Scr was significantly higher in UUO rats compared to sham-operated rats and was lower in enalapril group and DBT group, compared with UUO group (b). There was no difference between enalapril-treated rats and DBT-treated rats. Data are represented as mean ± SD. *n* = 8. ^*∗*^ *p* < 0.01 versus sham group. ^A^ *p* < 0.05 versus UUO group.

We observed that rats with UUO had obviously increased blood urea nitrogen (BUN) compared with sham-operated rats (sham 6.96 ± 1.47 mmol/L, UUO 11.55 ± 2.78 mmol/L, enalapril 9.23 ± 1.75 mmol/L, and DBT 8.92 ± 1.90 mmol/L, resp., *p* = 0.003) (Figure 1(a)). Serum creatinine (Scr) level was also higher in rats with UUO compared with sham-operated group (sham 50.94 ± 6.82 *μ*mol/L, UUO 75.99 ± 13.65 *μ*mol/L, enalapril 66.50 ± 7.38 *μ*mol/L, and DBT 60.60 ± 9.34 *μ*mol/L, resp., *p* = 0.001) (Figure 1(b)). Levels of BUN and Scr decreased in enalapril group and DBT group, with statistical significance. These data suggested that enalapril and DBT had renoprotective effect on UUO rats. However, there were no significant differences in the levels of Scr (*p* = 0.972) and BUN (*p* = 0.868) between enalapril and DBT groups, but DBT possessed a better renal protective effect than enalapril on UUO rats on numerical level.

### 3.2. DBT Attenuated Tubulointerstitial Fibrosis in Rats with UUO

**Figure 2:**
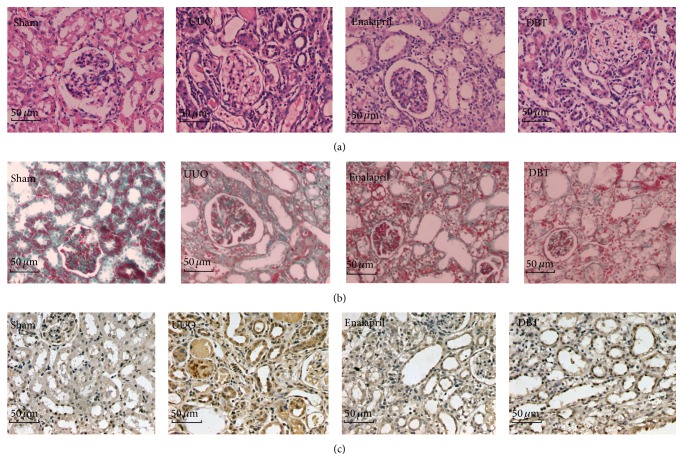
DBT attenuated tubulointerstitial injury, extracellular matrix (ECM) deposition, and expression of Col I in the obstructive kidneys after UUO. Tubulointerstitial injury in the obstructive kidneys (HE staining of rats' kidney sections, ×400). Tubular dilatation, tubular atrophy, tubular epithelial cells vacuolization, interstitial inflammatory cells infiltration, and interstitial fibrosis were observed in rats with UUO. Treatment with DBT and enalapril attenuated these changes (a). Deposition of ECM in the obstructive kidneys (Masson staining of rats' kidney sections, ×400). Rats in UUO group showed increased deposition of ECM. Treatment with DBT and enalapril attenuated these changes (b). Col I deposition in the obstructive kidneys (immunohistochemistry, in brown, ×400). Rats in UUO group showed an obvious Col I staining. Treatment with DBT and enalapril attenuated these changes (c).

**Figure 3:**
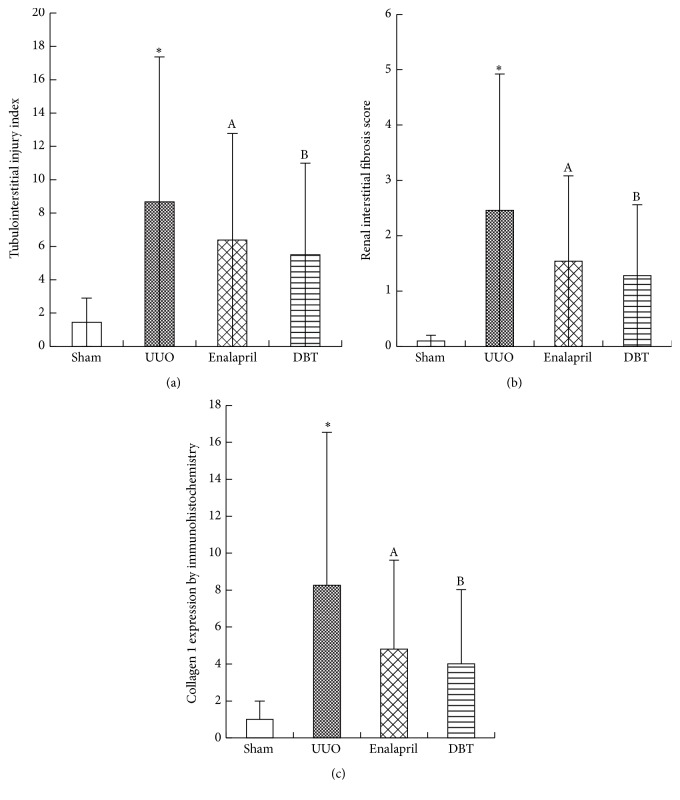
Tubulointerstitial injury index, renal interstitial score, and relative expression of Col I in the obstructive kidneys. UUO group exhibited a significant increasing in tubulointerstitial injury index (a), interstitial fibrosis score (b), and staining positive area of Col I (c). Treatment with DBT and enalapril attenuated all the scores. Tubulointerstitial injury index, interstitial fibrosis score, and the staining positive area of Col I were all lower in DBT group than in enalapril-treated group. All data were presented as means ± SD, *n* = 8 (^*∗*^ *p* < 0.05 versus sham group; ^A^ *p* < 0.05 versus UUO group; and ^B^ *p* < 0.05 versus enalapril group).

To evaluate the effect of DBT on renal fibrosis, we treated UUO rats with DBT (9 g/kg/d) and enalapril (10 mg/kg/d). Renal tissue from UUO rats developed TIF, which was manifested by tubular dilatation, tubular atrophy, tubular epithelial cells vacuolization, interstitial inflammatory cells infiltration, and interstitial fibrosis. DBT 9 g/kg/day treatment significantly attenuated tubulointerstitial injury at 14 days after UUO, as compared to untreated UUO rats (5.50 ± 1.20 versus 8.68 ± 1.03). The enalapril treatments also attenuated the renal pathological alterations and reduced tubulointerstitial injury scores (6.39 ± 1.18 versus 8.68 ± 1.03). Notably, tubulointerstitial injury index (*p* = 0.037) and interstitial fibrosis score (*p* = 0.012) in the DBT group were significantly lower than those in the enalapril group (Figures 2(a), 2(b), 3(a), and 3(b)).

**Figure 4:**
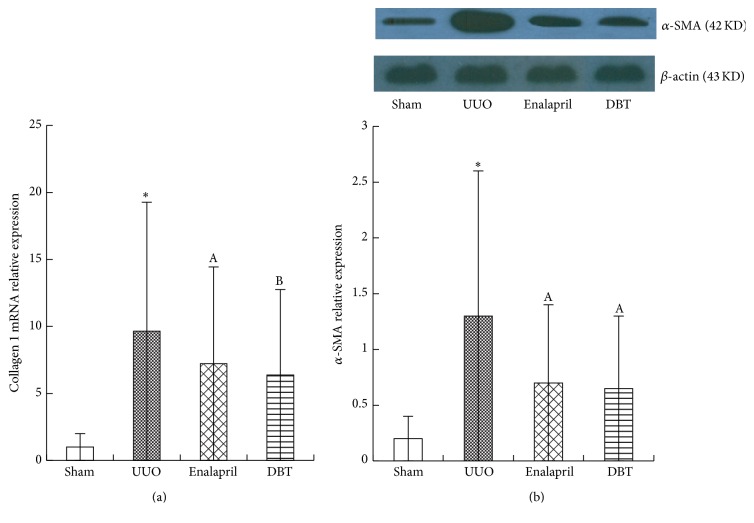
BDT decreased Col I mRNA levels in the obstructive kidneys after UUO at 14 days (a). Col I mRNA abundance was determined by real-time PCR. Col I mRNA levels were normalized to actin and expressed as the fold change in reference to Col I mRNA in sham-operated rats. Expression of Col I mRNA was upregulated in UUO group; DBT and enalapril treatment downregulated the expression of Col I mRNA in DBT group and enalapril group (*p* = 0.000). The level of Col I mRNA was lower in DBT-treated group than in enalapril group (a). DBT decreased the protein expressions of *α*-SMA in the obstructed kidneys as shown by Western blot (b). The column section indicated the relative expression level of *α*-SMA in the obstructive kidneys in each group. The expression was higher in UUO group than in sham group. Treatment with DBT and enalapril reduced the level of *α*-SMA protein. There was no difference between DBT and enalapril groups. All data were presented as means ± SD, *n* = 8 (^*∗*^ *p* < 0.05 versus sham group; ^A^ *p* < 0.05 versus UUO group; and ^B^ *p* < 0.05 versus enalapril group).

Collagen I (Col I) is the main ECM component which accumulates in the kidney during TIF. Next, the mRNA/protein expression of Col I was assessed by immunohistochemistry staining and real-time PCR. DBT induced a significant reduction of mRNA/protein Col I expression, compared with UUO group (mRNA: *p* = 0.000, protein: *p* = 0.000) (Figures 2(c), 3(c), and 4(a)). Compared with enalapril-treated rats, DBT treatment had an obvious influence on the mRNA and protein expression of Col I, significantly (mRNA: *p* = 0.040, protein: *p* = 0.047).

Renal interstitium fibroblasts are the essential source of fibrotic matrix. *α*-smooth muscle actin (*α*-SMA) is a marker of fibroblast activation. We observed that DBT significantly reduced the protein expression of *α*-SMA, compared with UUO group (*p* = 0.002) (Figure 4(b)). However, there was no difference between enalapril and DBT group (*p* = 0.757).

### 3.3. DBT Attenuated NLRP3 Inflammasome Expression in the Obstructive Kidney after UUO

**Figure 5:**
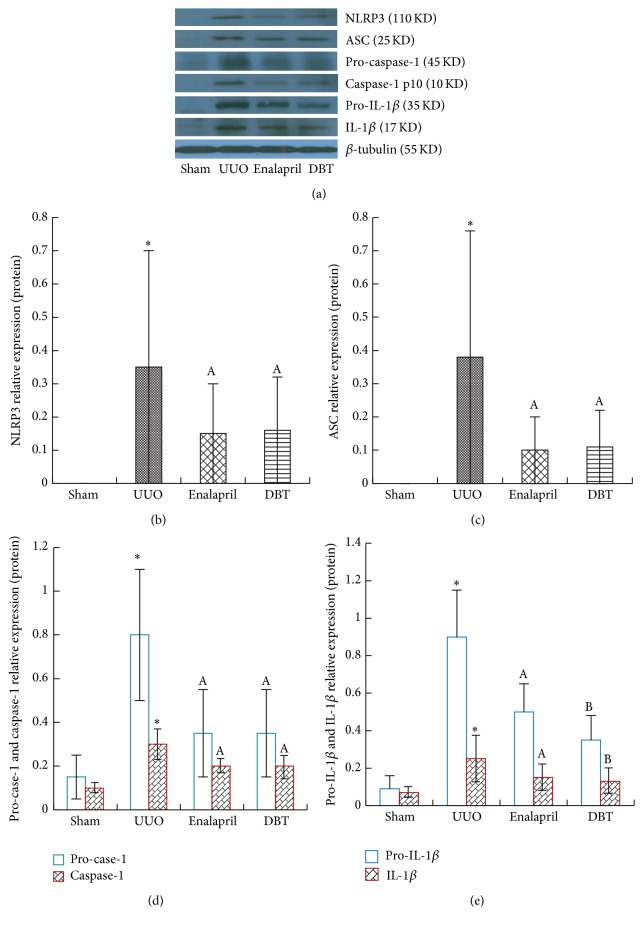
DBT inhibited the expression of NLRP3, ASC, pro-caspase-1, caspase-1, pro-IL-1*β*, and IL-1*β* in UUO rats by Western blot. UUO rats were given DBT and enalapril for treatment, and expressions of NLRP3, ASC, pro-caspase-1, caspase-1, pro-IL-1*β*, and IL-1*β* were measured by Western blot (a). Typical results are shown. In UUO group, NLRP3, ASC, pro-caspase-1, caspase-1, pro-IL-1*β*, and IL-1*β* were all significantly elevated. DBT and enalapril treatment decreased their expression (b, c, d, e). There were no differences between DBT and enalapril group, except for the expression of pro-IL-1*β* and IL-1*β*. DBT showed better significant effects on inhibiting the expression of pro-IL-1*β* and IL-1*β* compared to enalapril (e). All data were presented as means ± SD, *n* = 8 (^*∗*^ *p* < 0.05 versus sham group; ^A^ *p* < 0.05 versus UUO group; ^B^ *p* < 0.05 versus enalapril group).

**Figure 6:**
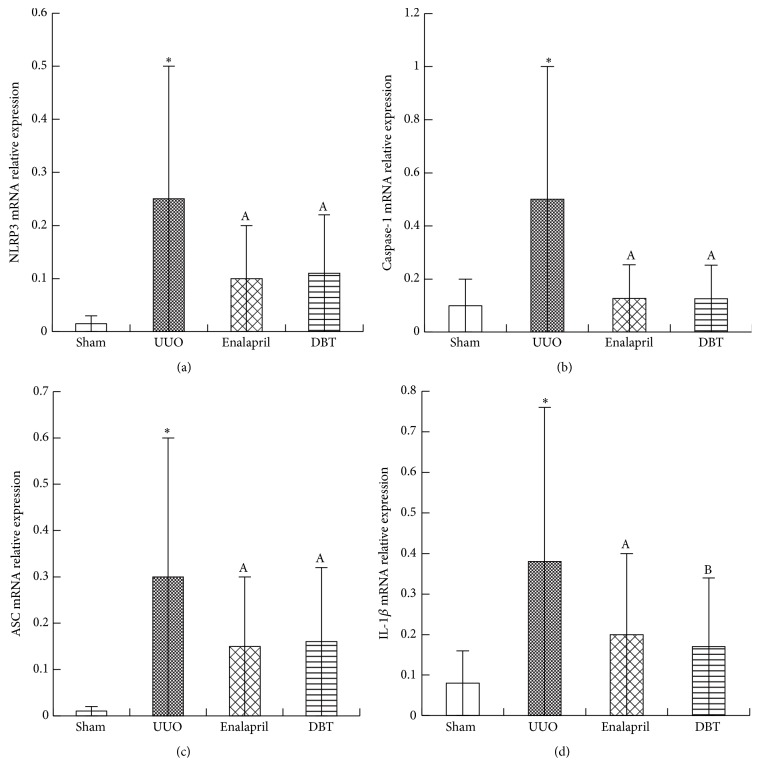
DBT inhibited the mRNA expression of NLRP3, ASC, caspase-1, and pro-IL-1*β* in UUO rats by real-time PCR. Typical results are shown. In UUO group, NLRP3, ASC, caspase-1, and IL-1*β* were all significantly elevated. DBT and enalapril treatment decreased their expression (a, b, c, d). There were no differences between DBT and enalapril group, except for the expression of IL-1*β* mRNA. DBT showed better significant effects on inhibiting the expression of pro-IL-1*β* mRNA compared to enalapril (d). All data were presented as means ± SD, *n* = 8 (^*∗*^ *p* < 0.05 versus sham group; ^A^ *p* < 0.05 versus UUO group; and ^B^ *p* < 0.05 versus enalapril group).

Since that DBT could effectively attenuate interstitial fibrosis induced by UUO, the influence of DBT on NLRP3 inflammasome expression was then observed in the obstructive kidney. The differences in NLRP3, ASC, caspase-1, and IL-1*β* expressions between the four groups were confirmed by protein and mRNA analysis as determined by Western blot (Figure 5) and real-time PCR (Figure 6), respectively. In untreated UUO rats, the protein expressions of NLRP3 (*p* = 0.001), ASC (*p* = 0.001), pro-caspase-1 (*p* = 0.000), caspase-1 (*p* = 0.031), pro-IL-1*β* (*p* = 0.002), and IL-1*β* (*p* = 0.020) were dramatically upregulated, along with the mRNA expression of NLRP3 (*p* = 0.002), ASC (*p* = 0.003), caspase-1 (*p* = 0.001), and IL-1*β* (*p* = 0.003). Treatment with enalapril and DBT significantly reduced these inflammatory cytokines' expression. There were no significant differences between enalapril and DBT group, except for the expression of pro-IL-1*β* (*p* = 0.030), IL-1*β* (*p* = 0.044), and IL-1*β* mRNA (*p* = 0.032) (Figures 5(a), 5(e), and 6(d)).

### 3.4. DBT Attenuated Inflammatory Cytokine IL-1*β* Kidney Homogenates after UUO

**Figure 7:**
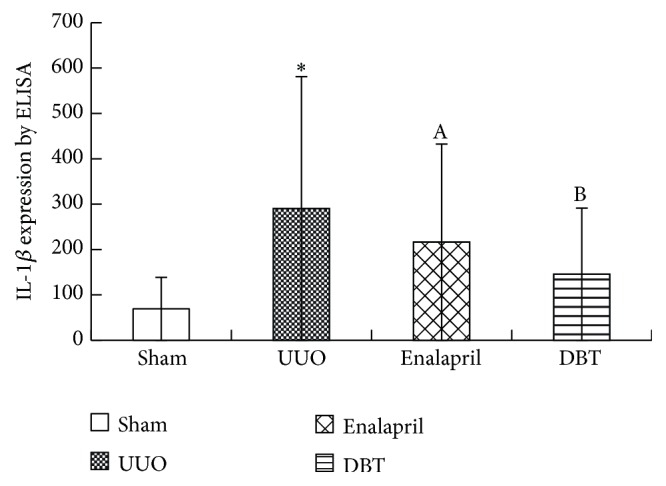
IL-1*β* expression in obstructive kidney in UUO rats by ELISA. IL-1*β* expression was quantified using ELISA. IL-1*β* protein was expressed as pg per mL of total protein (pg/mL total protein). Compared with sham group, IL-1*β* protein expressions were dramatically upregulated in UUO rats. Treatment with enalapril and DBT significantly ameliorated IL-1*β* expression (Figure 7). There was a significant difference between enalapril and DBT group (*p* = 0.021). The data were presented as means ± SD, *n*  =  8. (^*∗*^ *p* < 0.05 versus sham group; ^A^ *p* < 0.05 versus UUO group; and ^B^ *p* < 0.05 versus enalapril group).

For further conformation of cytokine IL-1*β* expression, kidney homogenates were analyzed for IL-1*β* using specific ELISA kits from R&D Systems according to the manufacturer's instructions. Compared with sham group, IL-1*β* protein expressions were dramatically upregulated in untreated UUO rats (sham 69.25 ± 18.91 pg/mL versus UUO 290.56 ± 40.32 pg/mL, *p* = 0.001). Treatment with enalapril and DBT significantly ameliorated the expression of inflammatory cytokine IL-1*β*, compared with UUO group (enalapril 216.31 ± 30.56 pg/mL and DBT 145.72 ± 32.44 pg/mL, *p* = 0.000) (Figure 7). There was a significant difference between enalapril and DBT group (enalapril 216.31 ± 30.56 pg/mL and DBT 145.72 ± 32.44 pg/mL, *p* = 0.021). DBT can substantially improve IL-1*β* expression in obstructive renal tissue.

## 4. Discussion

Nonmicrobacillary inflammation is an important component of many acute and chronic renal diseases [32]. Renal tubular epithelial cells injuries were due to a variety of insults, including ischemia, obstruction, and immune-mediated mechanisms [33], which can result in the release of endogenous cellular components and activate the NLRP3 inflammasome in rats with UUO [34]. NLRP3 inflammasomes regulate the production of proinflammatory cytokines, such as IL-1*β* and IL-18. The ability of NLRP3 inflammasome to respond to a variety of endogenous danger signals via a single common pathway makes it an attractive therapeutic target for CKD.

UUO is recognized as an ideal experimental model of renal interstitial fibrosis. Ureteral obstruction leads to a significant inflammatory and fibrotic response, followed by infiltration of inflammatory cells, renal interstitial myofibroblast proliferation, and ECM accumulation in the renal interstitium. Vilaysane et al. documented that there was increased NLRP3 mRNA/protein expression in UUO mice as well as in tubular epithelial cells (TECs) [35]. Fang et al. reported that proteinuria could induce NLRP3 inflammasome activation in TECs through endoplasmic reticulum stress [36]. These studies suggested that intrinsic renal cells might be involved in the activation of the NLRP3 inflammasome, and the activation of the NLRP3 inflammasome might be an important mechanism for the development of tubulointerstitial inflammation irrespective of the initial cause.

Nonetheless, the role of the NLRP3 inflammasome during ureteral obstruction remains controversial, Pulskens et al. found that Nlrp3 prevents early renal interstitial edema and vascular permeability in unilateral ureteral obstruction before 14 days after UUO surgery, they attribute that to the noncanonical effect of Nlrp3 [37]. Shigeoka et al. reported that reduced renal I/R injury was observed in mice deficient for NLRP3, but not ASC or caspase-1; they concluded that NLRP3-dependent, inflammasome-independent pathway may contribute to the development of I/R injury in the kidney [38].

In the present study, we showed that UUO rats displayed renal interstitial injury and fibrosis after 14 days of surgery, characterized by relatively increasing levels in serum creatinine, elevated tubulointerstitial injury index and score of tubulointerstitial fibrosis, and high expression of Col I mRNA/protein and *α*-SMA in obstructive renal tissues. Meanwhile, NLRP3 inflammasome activity presented in the UUO rats as indicated by the appearance of ASC, cleaved IL-1*β*, and active caspase-1 subunits (Casp1-p10). The mRNA and protein levels of NLRP3, ASC, caspase-1, and IL-1*β* were promoted in UUO group. Activation of NLRP3 inflammasome was accompanied by the process of fibrosis. DBT can obviously ameliorate the activation of NLRP3 inflammasome by decreasing the mRNA and protein levels of NLRP3, ASC, caspase-1, and IL-1*β*. The renoprotective and antifibrotic effects of DBT are connected with its negative impact on the activation of NLRP3 inflammasome after UUO surgery.

Compared with Pulskens et al. study [37], our work was conducted on the 14 days after UUO surgery. Interestingly, after 14 days, no differences in renal injury could be found anymore as tubular injury was at the maximum. We speculated that the noncanonical effect of Nlrp3 prevents tubular epithelial cells and vascular permeability only in early stage (before 14 days after UUO surgery) and did not alter the progression of fibrosis after UUO.

According to traditional Chinese medicine (TCM) theory, renal fibrosis is caused by a dual deficiency of Qi-yin and blood stasis. DBT had good functions of nourishing Qi (energy flow) and enriching Xue (blood), as well as resolving Yuxue (blood stasis) [39]. DBT is composed of RAS and RA with a weight ratio of 1 : 5. The weight ratio of RAS and RA was on a strict scientific basis. The complete functions of RA require the assistance of RAS to achieve the maximal therapeutic purposes [40, 41]. Significantly, higher amounts of angelica root-derived ferulic acid and Z-ligustilide and astragalus root-derived astragaloside IV, calycosin, and formononetin were found in DBT in a 1 : 5 ratio [42], in which two major constituents, astragaloside IV (AS-IV) and ferulic acid (FA), possessed the antifibrotic effects [43]. At the same time, AS-IV and FA had anti-inflammatory effect. A study of Wang B showed that macrophages were one of AS-IV targeted cells. AS-IV inhibited IL-1*β*, TNF-*α*, and nitric oxide (NO) and protected against IL-1*β*-induced joint inflammation and cartilage destruction in adjuvant-induced arthritis (AIA) rats [44]. The study of Li et al. also confirmed that the anti-inflammatory mechanism of AS-IV was associated with inhibiting production of TNF-*α*, IL-1*β*, and nuclear factor-*κ*B (NF-*κ*B) in cerebral injury induced by ischemia/reperfusion (I/R) [45]. FA* per se* is an antioxidant and anti-inflammatory agent derived from plants. Huang et al. found that pretreatment with FA significantly inhibited production of TNF-*α* and IL-1*β* in Alzheimer's disease [46]. Roy et al. revealed a significant reduced expression of TGF-*β*1 and IL-1*β* in the pancreatic *β*-cell of FA-treated diabetic rats [47]. FA decreased the hydrogen peroxide-induced IL-1*β* and TNF-*α* gene expression in chondrocytes [48]. All of these studies suggested that AS-IV and FA had an obvious effect on suppressing IL-1*β* expression.

The maturation of proinflammatory cytokines IL-1*β* was mainly caused by NLRP3 inflammasome formation and activated caspase-1 in response. Our data demonstrated that DBT can decrease IL-1*β* mRNA/protein level and attenuate TIF by interference with NLRP3 inflammasome expression in DBT-treated rats. Compared with enalapril-treated rats, DBT can significantly decrease IL-1*β* expression. That may be partly due to anti-inflammatory effect of Z-ligustilide, a component of RAS. Ma and Bai found that Z-ligustilide significantly inhibited NF-*κ*B activation and the production of IL-1*β* and TNF-*α* in experimental ovariectomized (OVX) osteopenic rats and endotoxin-induced uveitis rats [49, 50]. Generally speaking, FA and Z-ligustilide, two major compounds in RA, activated the Nrf2 pathway and decreased NF-*κ*B luciferase activity, which may contribute to the anti-inflammatory activity of RA [51].

Except for anti-inflammation, DBT can inhibit the synthesis of extracellular matrix and balance the MMP/TIMP-1 system. Gao et al. investigate the antifibrotic effects of DBT on bleomycin-induced pulmonary fibrosis in rats and found that DBT administration attenuated the degree of alveolitis and lung fibrosis and markedly reduced the mRNA levels of matrix metalloproteinase-9 (MMP-9) and tissue inhibitor of metalloproteinase-1 (TIMP-1) [27]. Xu et al. demonstrated that administration of FA significantly decreased the levels of collagen and tissue inhibitor of metalloproteinases (TIMPs) and positively modulated the expression of matrix metalloproteinases (MMPs) in liver fibrosis induced by alcoholic and polyunsaturated fatty acid [52]. These results proved that the mechanisms of multitargets of traditional Chinese medicine can be better used for antifibrotic treatment.

In this study, we also demonstrated that enalapril could significantly attenuate the tubulointerstitial expression of the NLRP3 inflammasome and renal interstitial fibrosis in UUO rats. Surprisingly, DBT treatment was better compared to enalapril in UUO rats. The tubulointerstitial injury index, tubulointerstitial fibrosis score, and collagen 1 mRNA/protein expression were lower in DBT group than in enalapril group. But there were no obvious differences in *α*-SMA expression and the mRNA/protein expression of NLRP3 and ASC and caspase-1 between enalapril and DBT group. These data show that enalapril and DBT have the equivalent effect on suppressing the activation of fibroblasts and NLRP3 inflammasome in UUO rats. However, the overall antifibrotic function of enalapril was inferior to DBT. Traditional Chinese medicines are large complex compounds, which possess multiple target actions and multiple mechanisms. Such as DBT, the multiple target actions and latent multiple mechanisms contributed to the better antifibrotic function.

In summary, the results of the present study clearly show that DBT manifested significant renoprotective effects. DBT can attenuate the UUO-induced TIF. The mechanisms underlying this protective action may be attributed to modulating NLRP3 activated level and then decreasing cytokine IL-1*β* secretion. These findings suggest that DBT may be a novel potential candidate to improve TIF. However, more studies need to be conducted to clarify the exact antifibrotic mechanism of DBT.
